# Aloperine targets lysosomes to inhibit late autophagy and induces cell death through apoptosis and paraptosis in glioblastoma

**DOI:** 10.1186/s43556-023-00155-x

**Published:** 2023-11-17

**Authors:** Ting Tang, Hui Liang, Wuting Wei, Yanling Han, Liang Cao, Zixiang Cong, Shiqiao Luo, Handong Wang, Meng-Liang Zhou

**Affiliations:** 1 Department of Neurosurgery, Xuanwu Hospital Capital Medical University, Beijing, P.R. China; 2 Department of Neurosurgery, Affiliated Jinling Hospital, Medical School of Nanjing University, Nanjing, P.R. China; 3 Department of Neurosurgery, The First Affiliated Hospital, Guangxi Medical University, Nanning, P.R. China; 4 Department of Medical Oncology, Affiliated Chuzhou Hospital of Anhui Medical University, The First People’s Hospital of Chuzhou, Chuzhou, P.R. China; 5 Department of Neurosurgery, Affiliated Jinling Hospital, Nanjing Medical University, Nanjing, P.R. China; 6 Department of Neurosurgery, Benq Medical Center, Nanjing Medical University, Nanjing, China

**Keywords:** Aloperine, Glioblastoma, Autophagy, Lysosome, Paraptosis

## Abstract

Glioblastoma (GBM) is an aggressive intracranial tumour, and current chemotherapy regimens have limited efficacy. Aloperine (ALO), a natural alkaline compound, has shown potential as an antitumor agent. However, the effect of ALO against GBM remains unclear. This study aimed to investigate the function of ALO in treating GBM. U87, A172, and GL261 cell lines were used for in vitro experiments, and GL261 was also used to establish in vivo models. The results showed that ALO inhibited the proliferation of GBM cells by cell cycle arrest and apoptosis. Furthermore, autophagy was found to play a critical role, suggested by observation of autophagosomes under the transmission electron microscopy. It was discovered for the first time that ALO targeted lysosomes directly in glioma cells, tested by fluo-rescence-labelled ALO and organelle-localizing probes. In addition, ALO inhibited late autophagy and induced paraptosis in GBM, verified by classical gene expression changes in qPCR and western blotting. Also, ALO inhibited tumour growth and acted synergistically with temozolomide in intracranial glioma mice models in vivo. Our findings suggest that ALO targets lysosomes to inhibit late autophagy in GBM, inducing cell cycle arrest, paraptosis, and apoptosis. ALO may therefore be a promising therapeutic agent for the treatment of GBM.

**Supplementary Information:**

The online version contains supplementary material available at 10.1186/s43556-023-00155-x.

## Introduction

Glioblastoma (GBM) is a highly aggressive malignancy of the central nervous system. The current standard of care for GBM involves surgical resection, followed by radiotherapy and temozolomide (TMZ) chemotherapy [1, 2]. However, the development of resistance to TMZ remains a significant obstacle in the treatment of GBM [3]. And till now, there is no other effective medicine for first-line chemotherapy in GBM yet. Therefore, there is an urgent need to develop new and effective therapeutic strategies for GBM.

Aloperine (ALO) is a natural alkaline compound extracted from Sophora alopecuroides Linn [4]. It has been reported that ALO and its derivatives are involved in various biological activities [4, 5], performing anticancer [6, 7], antiviral [8], anti-arrhythmic [9], anti-ischemia/reperfusion injury [10], and anti-inflammatory [11–14] effects. ALO has been reported to induce cell cycle arrest [15–17], autophagy [18, 19], and apoptosis [6, 20, 21] in various cancers, including lung cancer [7, 22], hepatocellular carcinoma [16], colon cancer [17], prostate cancer [15], ovarian cancer [23], thyroid cancer [21], and multiple myeloma [6]. Cell cycle arrest and apoptosis are the mechanisms by which ALO kills tumours with the highest frequency shown in the previous research. In tumour treatment, reactive oxygen species (ROS) accumulation causing severe oxidative stress is a sharp weapon. ALO has also been reported to affect the production of ROS [14]. It may be the key mechanism that ALO kills tumours through ROS production.

Recent studies have demonstrated that ALO induces apoptosis in glioma cells in vitro, including SK-N-AS and U118 cells, and the Bcl2 protein, an apoptosis regulator, was predicted to be a target of ALO [24]. However, the antitumor effects of ALO on other glioma cells in vitro and in vivo, as well as its underlying mechanisms, have not been fully elucidated.

In this study, we explored the antitumor activity of ALO on GBM and identified ALO-regulated cell death pathways based on both morphological changes in organelles and previous research reports. In particular, the target of ALO was identified to further explain the mechanism of ALO action.

## Results

### ALO inhibited GBM cell proliferation and induced cell cycle arrest in vitro

**Fig. 1:**
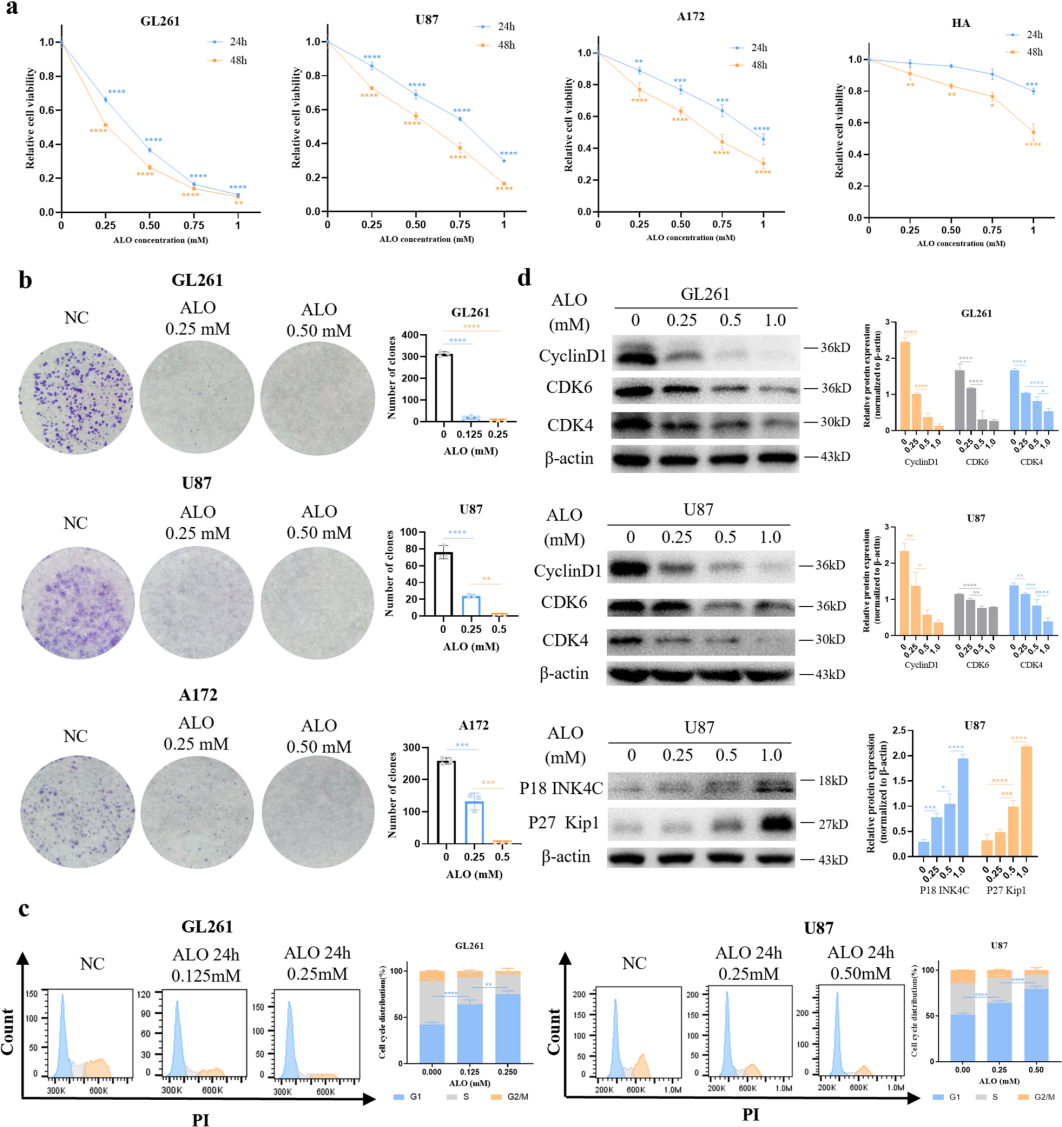
ALO inhibited glioma cells proliferation and induced cell cycle arrest in vitro.** a** Cell viability of GL261, U87, A172, and HA exposed to ALO for different concentrations and time courses by cck-8 test (*n* = 4). **b** Clone forming ability of GL261, U87, and A172 single cell after ALO treatment by clone forming assay (*n* = 3). **c** Cell cycle distribution of GL261 and U87 exposed to ALO by flow cytometry (*n* = 3). **d** Expression of G1-phase cell cycle related proteins and cell cycle inhibitors with ALO in GL261 and U87, tested by western blot (*n* = 3). Data are presented as mean ± SD, *p < 0.05, **p < 0.01, ***p < 0.001, ****p < 0.0001. ALO, aloperine; HA, normal human astrocyte; NC, negative control

The treatment of increasing concentrations (0–1.0 mM) of ALO for 24 h led to a decrease in the cell survival rate, with GL261 cells showing the highest sensitivity, followed in descending order by U87 and A172 (Fig. 1a). However, the normal HA cell line was much more insensitive to ALO, with only a slight decrease in cell viability when treated with 1.0 mM ALO (Fig. 1a). The half-maximal inhibitory concentration (IC_50_) values of ALO were 260.6 µM, 733.3 µM, 960.9 µM, and 1710 µM, for the GL261, U87, A172, and HA cell lines, respectively. When the treatment time was extended to 48 h, only a slightly greater killing effect of ALO was observed in all these cells. The clone formation assay revealed that 0.5 mM ALO inhibited the clone-forming ability of GL261 and U87 single cell completely, as well as that of A172 (Fig. 1b), which was not as sensitive to ALO as the other cells, as indicated by the cck-8 assay.

The treatment of GL261 and U87 cells with ALO at low concentrations (0.125 mM and 0.25 mM, respectively) for 24 h resulted in the arrest of the cell cycle at the G1 phase, as indicated by flow cytometry analysis (Fig. 1c). The reduction in ALO concentration was accompanied by a decrease in the expression of cyclin D1, CDK6, and CDK4, representative proteins of the G1 phase (Fig. 1d). Additionally, the expression of cell cycle inhibitors such as P18^INK4C^ and P27^Kip1^ was decreased in U87 (Fig. 1d).

### ALO induced apoptosis but not necroptosis in GBM cells in vitro

**Fig. 2:**
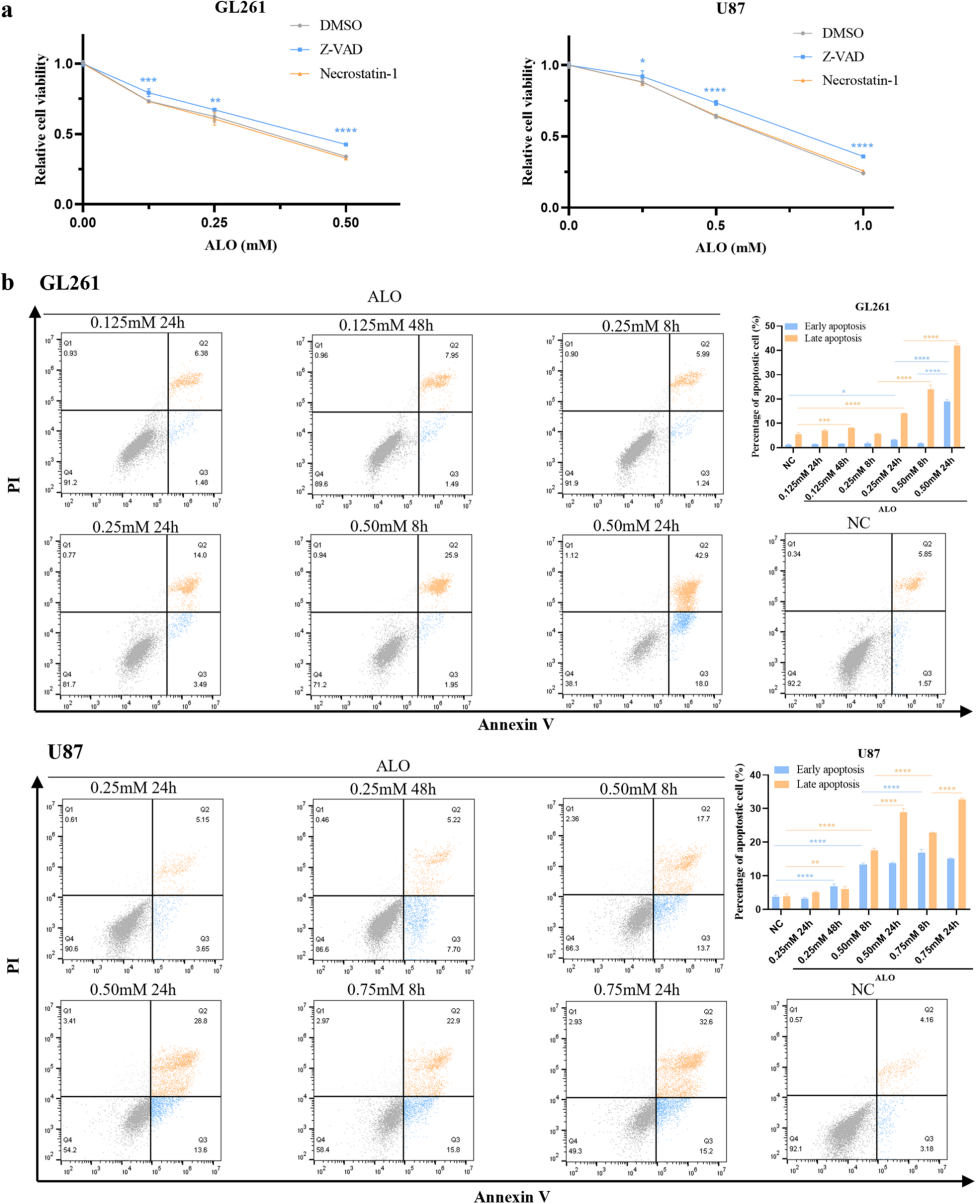
ALO induced cell apoptosis in GBM cells in vitro. **a** Cell viability of GL261 and U87 exposed to ALO, pretreated with necrosis inhibitor or apoptosis inhibitor by cck-8 test (*n* = 4). **b** Cell apoptosis of GL261 and U87 with ALO treatment for different concentrations and time courses by flow cytometry (*n* = 3). Data are presented as mean ± SD, *p < 0.05, **p < 0.01, ***p < 0.001, ****p < 0.0001. ALO, aloperine; NC, negative control

In vitro, ALO induced apoptosis but not necroptosis in GBM cells. Partial attenuation of cell death was observed with the apoptosis inhibitor Z-VAD, but not with the necroptosis inhibitor necrostatin-1 (Fig. 2a), suggesting the critical function of apoptosis in GBM. However, low ALO concentrations triggered apoptosis only after a prolonged treatment time of 48 h, resulting in a low apoptosis rate. In contrast, a higher ALO concentration (0.25 mM for GL261 and 0.5 mM for U87) resulted in a significant increase in the apoptosis rate after only 8 h of treatment (Fig. 2b).

### ALO inhibited late autophagy in GBM cells in vitro

**Fig. 3:**
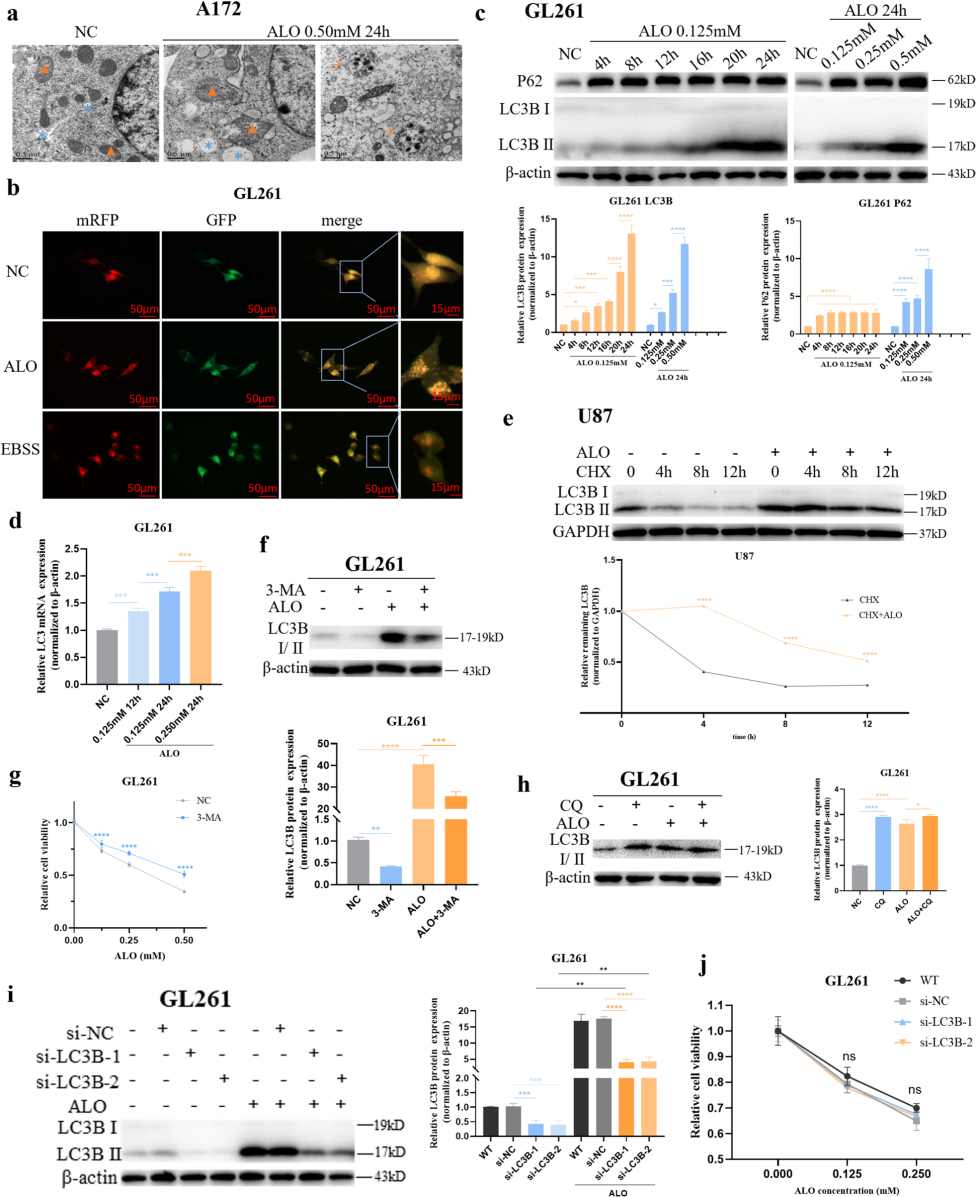
ALO inhibited late autophagy in glioma cells in vitro. **a** Changes of organelles in A172 after ALO treatment by transmission electron microscopy. Orange triangle, mitochondria; blue star, endoplasmic reticulum; orange arrow, autophagosomes. **b** Tracking of the autophagic flux following ALO treatment dynamically by introducing a tandem mRFP-GFP-LC3 adenovirus into GL261, with EBSS treatment group as the autophagic positive control. **c** Expression of autophagy-related proteins, LC3B and p62, exposed to ALO in GL261 tested by WB (*n* = 3). **d** Transcriptional expression of LC3B, after ALO treatment in GL261 tested by qPCR (*n* = 3). **e** Degradation rate of autophagy-related protein LC3B exposed to ALO with CHX in U87 tested by WB (*n* = 3). **f** Expression of LC3B by WB, exposed to PI3K inhibitor, 5 mM 3-MA, with or without ALO in GL261, to ensure the inhibitory effect on the autophagic pathway (*n* = 3). **g** Cell viability of GL261 exposed to ALO, pre-treated with 3-MA by cck-8 test (*n* = 4). **h** Protein expression of LC3B, exposed to 10 µM CQ, with or without ALO in GL261 tested by WB (*n* = 3). **i** LC3B protein was decreased effectively by si-RNA transfection in GL261, tested by WB (*n* = 3). **j** Cell viability of GL261 exposed to ALO by cck-8 when LC3B was downregulated (*n* = 4). Data are presented as mean ± SD, *p < 0.05, **p < 0.01, ***p < 0.001, ****p < 0.0001, ns means not significant. ALO, aloperine; NC, negative control; WB, western blot; CHX, cycloheximide; EBSS, Earle’s Balanced Salt Solution; CQ, Chloroquine; 3-MA, 3-Methyladenine; WT, wildtype

Transmission electron microscopy (TEM) analysis revealed that ALO induced the formation of primary autophagosomes in A172 cells, but without fusion with lysosomes (Fig. 3a). In addition, ALO treatment caused swelling and rupture of the endoplasmic reticulum and mitochondria (Fig. 3a). To track the autophagic flux, a tandem mRFP-GFP-LC3-expressing adenovirus was introduced into GBM cells. Results showed that the formation of autophagosomes was hindered after ALO treatment, as comparable numbers of GFP and RFP puncta were observed, with no apparent quenching of the GFP signal in lysosomes, compared to the autophagic positive control group triggered by EBSS (Fig. 3b and S1a). The expression of the marker protein LC3B increased significantly, but no degradation of P62 was observed (expression increased in GL261 and almost unchanged in U87), as assessed by WB (Fig. 3c and S1b). The mRNA expression of LC3B was enhanced to a certain degree (Fig. 3d and S1c), but was not consistent with the protein expression level. Additional experiments with exposure to CHX showed that the decreased degradation rate of the LC3B protein was shown after ALO treatment (Fig. 3e). Furthermore, pretreatment with an effective concentration of 3-MA for 2 h, to block upstream autophagy (Fig. 3f and S1d), significantly inhibited the effect of ALO (Fig. 3g and S1e). These results provided evidence for the critical role of the basic level of autophagy in ALO treatment.

In addition, 12 h treatment of 10 µM CQ, which targets proton pumps to block autophagolysosome degradation, led to increased LC3B levels in both GL261 and U87 cells, but this effect was not obvious when cells were cotreated with ALO (Fig. 3h and S1f). When LC3B expression was knocked down by short interfering RNA (siRNA), the accumulation of LC3B after ALO treatment was not fully reversed (Fig. 3i), and the killing effect of ALO was also not influenced (Fig. 3j), which suggested that the accumulation of LC3B was a result of ALO treatment, but not the cause of cell death. These results indicated an inhibition of the late-phase autophagic flux after ALO treatment.

ALO targeted lysosomes directly and weakened its acidic conditions in GBM cells in vitro.

**Fig. 4:**
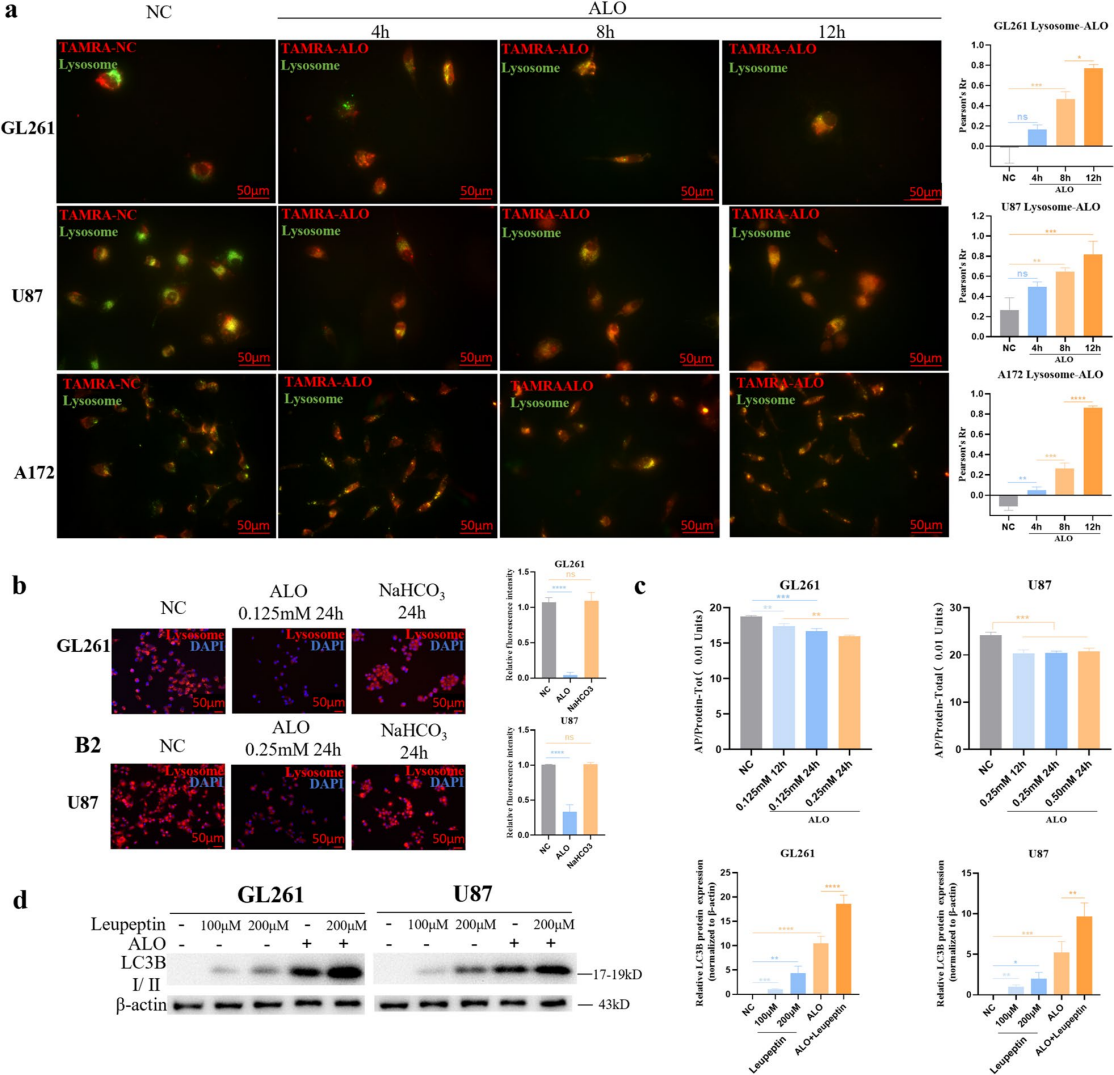
ALO targeted lysosomes directly and weakened its acidic conditions in GBM cells in vitro. **a** Tamra-se coupled ALO and lysosome-targeted fluorescent probe (green) colocalization analysis in GL261, U87, and A172 (*n* = 3). **b** Acidic conditions of lysosomes in GL261 and U87 exposed to ALO by lysosome-targeted fluorescent probe (red), with NaHCO_3_ treatment group as the negative control (*n* = 3). **c** Function of the acid phosphatase in lysosomes under conditions of cell lysis after the treatment of ALO in GL261 and U87. **d** Cell viability of GL261 and U87 exposed to ALO, pretreated with thiol protease inhibitor, Leupeptin, by cck-8 test (*n* = 4). Data are presented as mean ± SD, *p < 0.05, **p < 0.01, ***p < 0.001, ****p < 0.0001. ALO, aloperine; NC, negative control; WB, western blot

A fluorescent probe co-localization analysis with TAMRA-SE-coupled ALO treatment creatively confirmed that ALO targeted lysosomes (Fig. 4a), but not the ER (Fig. S2a) or mitochondria (Fig. S2b). Additionally, ALO attenuated the acidic conditions of lysosomes (Fig. 4b), as indicated by a decrease in the labelling of lysosomes by the acidophilic Lyso-Tracker. Moreover, no change in the acidic conditions of lysosomes was observed in the NaHCO_3_ treatment group (Fig. 4b), which indicated that the effect could be attributed to the unique pharmacological function of ALO, rather than the alkaline conditions per se. The functional inhibition of a classical enzyme in lysosomes, acid phosphatase, was minimal (although the reduction was statistically significant) when tested with an assay kit (P0326, Beyotime) under conditions of cell lysis after treatment with ALO (Fig. 4c). And also, even higher expression of LC3B was observed after pretreatment with the protease inhibitor of lysosomes, leupeptin, added to ALO treatment (Fig. 4d). However, the addition of leupeptin did not affect the killing effect of ALO (Fig. S2c). Based on this evidence, it was indicated that the particular target of ALO is probably the proton pumps on the membrane of lysosomes, rather than the acid hydrolases inside.

### ALO induced paraptosis in GBM cells in vitro

**Fig. 5:**
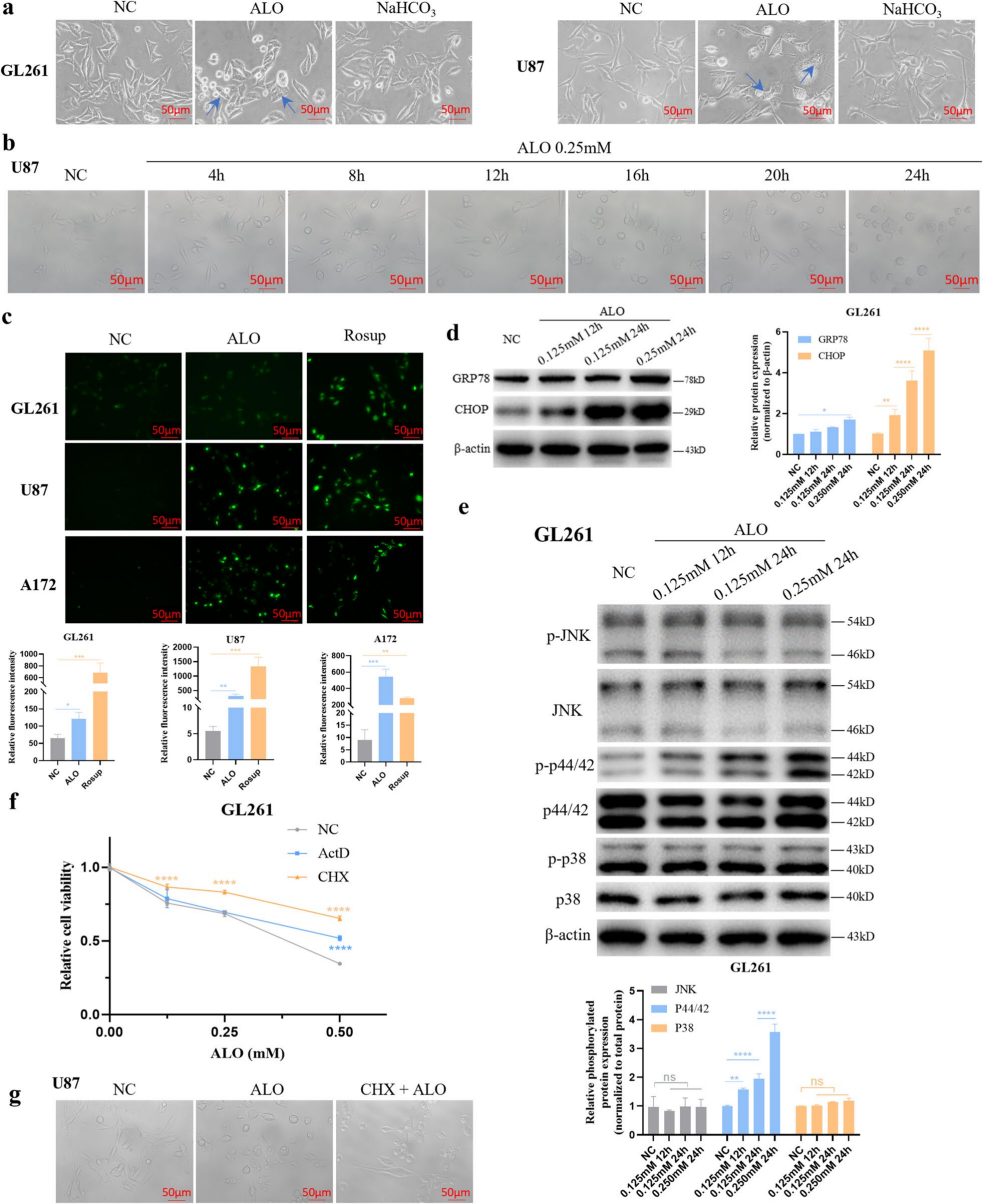
ALO induced paraptosis in GBM cells in vitro. **a** Cytoplasmic vacuolations were observed under the light microscope after ALO treatment in GL261 and U87. **b** Cytoplasmic vacuolations were observed under the light microscope after ALO treatment in U87. **c** ROS generation tested by the DCFH-DA probe in GL261, U87 and A172 was observed under the fluorescence microscope, with Rousup as the positive control (*n* = 3). **d** Expression of ER stress-related proteins exposed to ALO in GL261 tested by WB (*n* = 3). **e** Activation of MAPK pathways exposed to ALO in GL261 tested by WB (*n* = 3). **f** Cell viability of GL261 exposed to ALO by cck-8 test, pretreated with 2 µM ActD or 2 µg/ml CHX for 2 h (*n* = 4). **g** Cytoplasmic vacuolations were observed under the light microscope after ALO treatment in U87 with or without CHX pretreatment for 2 h. Data are presented as mean ± SD, *p < 0.05, **p < 0.01, ***p < 0.001, ****p < 0.0001, ns means not significant. ActD, Actinomycin D; ALO, aloperine; CHOP, C/EBP-Homologous Protein; CHX, cycloheximide; ER, endoplasmic reticulum; GRP78, 78 kDa Glucose-Regulated Protein; NC, negative control; WB, western blot

Cytoplasmic vacuoles were observed in GL261 and U87 cells under a light microscope after ALO treatment for 12 h at low concentration of 0.125 mM and 0.25 mM, respectively (Fig. 5a). Specifically, cytoplasmic vacuoles were formed in U87 cells as early as 8 h after ALO treatment (Fig. 5b). Vacuolation is a classic feature of paraptosis, and hence, other paraptosis-relevant changes were also assessed. Notably, ROS production increased (Fig. 5c), concomitantly with induced ER stress, which was indicated markedly by increased GRP78 (78 kDa glucose-regulated protein) and CHOP (C/EBP-homologous protein) protein levels according to WB analysis (Fig. 5d and S3a). The MAPK signalling pathway activity detection showed that the ERK (p44/42) and p38 pathways were activated by protein phosphorylation in both GL261 and U87 after ALO treatment (Fig. 5e and S3b). Furthermore, the gene transcription inhibitor ActD and the protein synthetization inhibitor CHX effectively reversed the cell-killing effect of ALO (Fig. 5f and S3c). Also, CHX treatment reduced the number of cytoplasmic vacuoles efficiently induced by ALO treatment (Fig. 5g and S3d). These results suggested that the vacuolation occurred due to an accumulation of newly-produced proteins.

### ALO inhibited tumour growth and exerted synergistic effects with TMZ in vivo

**Fig. 6:**
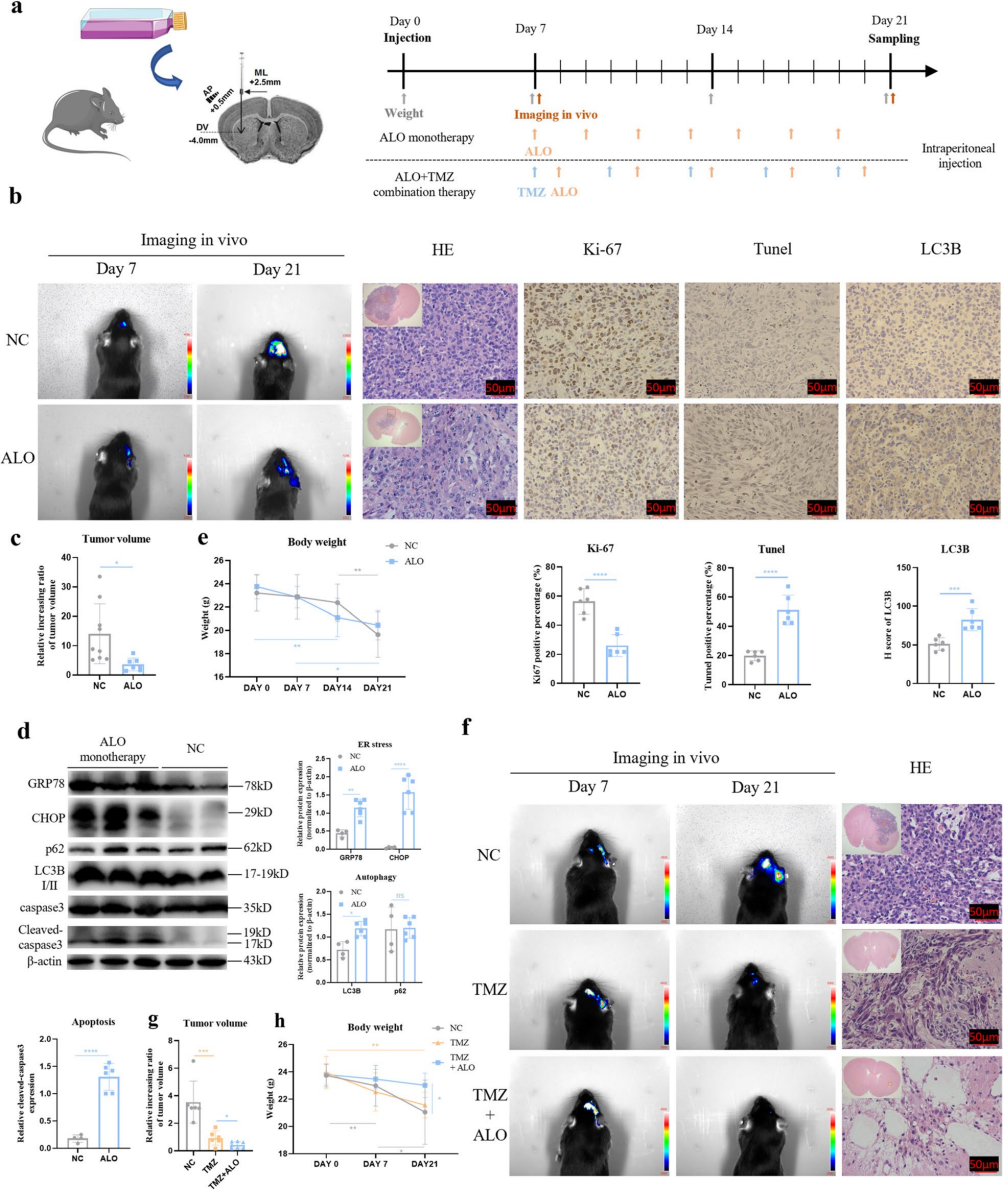
ALO inhibited the tumor growth and was synergistic with TMZ in vivo. **a** The intracranial tumour placement position model, and the timeline for modelling and treatment of ALO mono-therapy and TMZ + ALO combination therapy. **b-d** Living imaging (NC, *n* = 9; ALO, *n* = 6), weight change, and immunohistochemistry staining (NC, *n* = 6; ALO, *n* = 6) in ALO mono-therapy treatment. **e** Expression of endoplasmic reticulum stress-related proteins, autophagy-related proteins, and apoptosis-related protein in ALO mono-therapy treatment by western blot (NC, *n* = 4; ALO, *n* = 6). **f-h** Living imaging, body weight change, and HE staining in TMZ mono-therapy and TMZ + ALO co-therapy treatment (NC, *n* = 6; TMZ, *n* = 6; TMZ + ALO, *n* = 6). Data are presented as mean ± SD, *p < 0.05, **p < 0.01, ***p < 0.001, ****p < 0.0001, ns means not significant. AP, anteroposterior; ML, mediolateral; DV, dorsoventral; ALO, aloperine; TMZ, temozolomide; NC, negative control; HE, Hematoxylin-Eosin; GRP78, 78 kDa Glucose-Regulated Protein; CHOP, C/EBP-Homologous Protein

Intracranial stereotactic allograft glioma models were established with C57BL/6 mice to test the ALO treatment effect in vivo. The course of treatment is shown in Fig. 6a. The tumour volume was significantly reduced 21 days after 7 administrations of ALO monotherapy (Fig. 6b, c). Ki-67 staining indicated a lower proliferative rate of tumour cells in the ALO treatment group (Fig. 6b). TUNEL staining also indicated the apoptosis of tumour cells exposed to ALO (Fig. 6b). The LC3B expression level was increased significantly in the ALO treatment group, as shown by Immunohistochemistry (IHC) staining (Fig. 6b). It was also shown that autophagy and paraptosis were induced after ALO treatment by WB in vivo (Fig. 6d). This evidence was consistent with the results in vitro. However, although the tumour grew at a slower rate after ALO exposure, the tumour volumes were larger on day 21 than on day 7 (Fig. 6c). Additionally, the bodyweights of the mice decreased markedly after ALO treatment, especially during the early stage (Fig. 6e). Both of these outcomes indicated limitations of ALO monotherapy in vivo. Further application of combination therapy showed a synergistic effect of ALO and TMZ, with the tumour volumes decreased to the greatest extent in the combination therapy group (Fig. 6f, g), and the mouse body weights were stabilized (Fig. 6h).

**Fig. 7:**
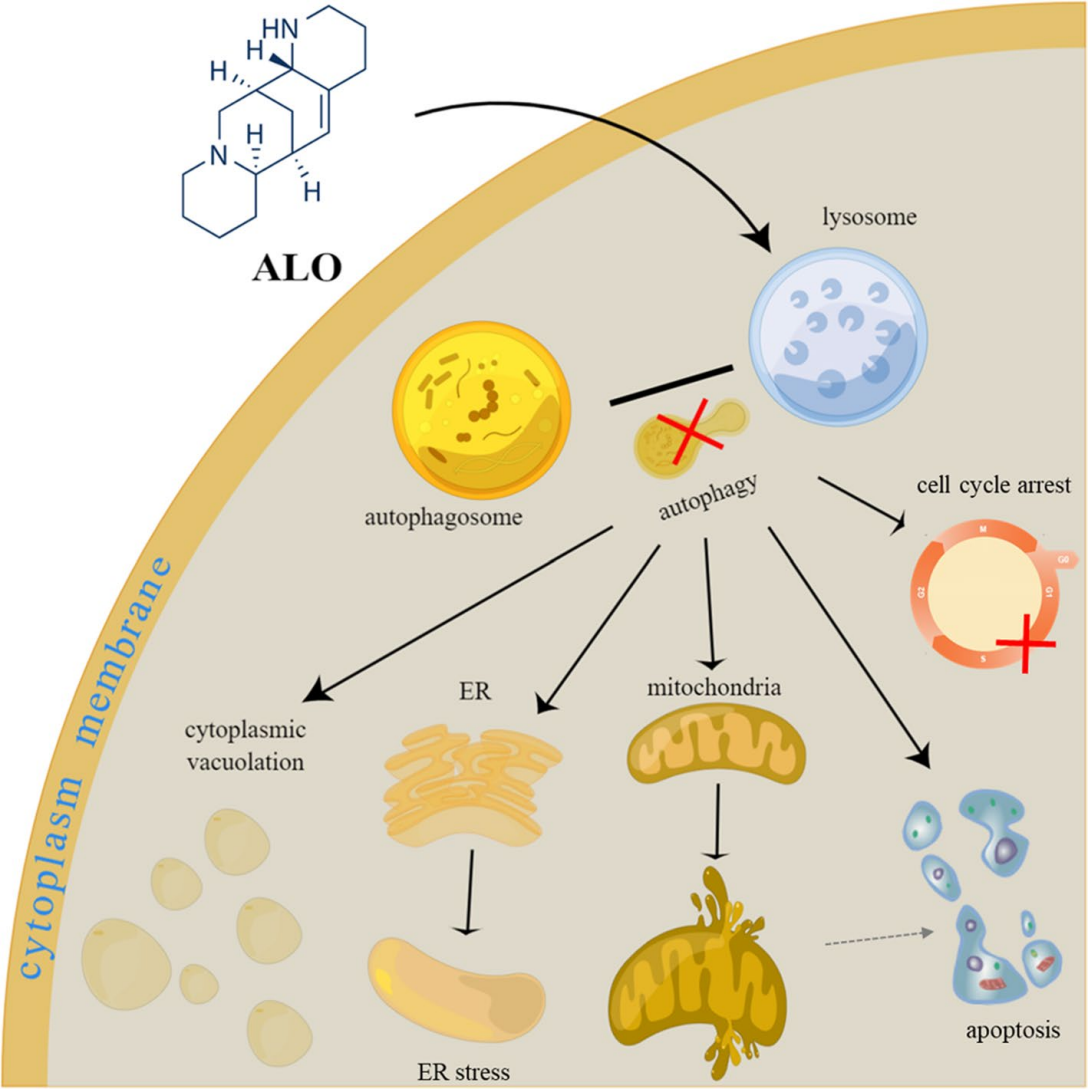
The schematic diagram shows the mechanism of ALO killing glioma cells. ALO targets lysosomes to inhibit late autophagy in GBM, inducing cell cycle arrest, paraptosis, and apoptosis. ALO, aloperine; ER, endoplasmic reticulum

The schematic diagram with the mechanism of ALO killing glioma cells in this study is shown in Fig. 7.

## Discussion

To the best of our knowledge, only one study has investigated the function of ALO treatment in glioma [24], and the role played by ALO in GBM remains incompletely understood. Our study aims to address this knowledge gap by demonstrating that ALO targets lysosomes and causes paraptosis in GBM. We also verified the effects of ALO on cell cycle arrest and apoptosis and revealed certain details of the mechanism. In summary, our work provides a better understanding of the mechanism of ALO action in GBM.

Our findings show that ALO inhibited GBM cell proliferation in vitro, with the sensitivity decreasing sequentially in GL261, U87, A172, and HA cells, as determined by the cck-8 assay. We suggest that ALO has potential value in treating GBM due to its greater killing effect on GBM cells than normal astrocytes. Furthermore, even at a low ALO concentration of 0.25 mM, we observed significant inhibition of clone formation, even in the A172 cell line, in which the IC50 was higher than that of the other cell lines.

In vivo experiments demonstrated that ALO monotherapy decreased the proliferation rate of intracranial glioma allograft cells. These experiments also indicated that ALO partially penetrated the blood-brain barrier in glioma models, which is a clear advantage in treating intracranial tumours. However, despite the inhibitory effect of ALO, the tumours continued to grow over time, even when the concentration and frequency of ALO were high enough to cause critical weight loss in mice. This may be related to the accumulation of ALO in the circulatory system and other tissues. Nevertheless, ALO performed well in combination with TMZ at a lower dose than that as a monotherapy, likely due to the combination of different lethal mechanisms. Since ALO application to tumours in vivo has rarely been reported, further research on the pharmacokinetics of ALO, an optimized dosing regimen, other therapy combinations, and survival analysis are needed. Also, glioma models using other cell lines including KR158 and human glioma stem cells may indicate the effect of ALO adequately.

Cell cycle arrest and apoptosis are among the common programmed cell death mechanisms induced by cancer chemotherapy. It has been reported that ALO causes cell cycle arrest in tumours, including lung cancer [18], hepatocellular carcinoma [16], colon cancer [17], and prostate cancer [15], in the G1/S [15, 18] or G2/M phase [16, 17]. In this study, we were the first to find that ALO induced G1/S cell cycle arrest in GBM by flow cytometry and WB. Since the phases of the cell cycle targeted by ALO were not consistent in the previous report [5], it was speculated that cell cycle arrest is likely a secondary effect of ALO, rather than the direct target. ALO was also indicated to induce apoptosis in many tumours [6, 16, 21], including in a recent report on glioma [24]. Yan and colleagues [24] identified the induction of apoptosis in SK-N-AS and U118 cell lines, as representatives of GBM cells, after treatment with ALO at 0.5 mM and 1.0 mM. The authors focused on apoptosis based on bioinformatics analysis, according to which Bcl2 was predicted as a target protein. However, there was no experimental evidence provided in the published article [24]. Additionally, no direct mechanism has been reported for any other tumours. In our study, the apoptosis-inducing effect of ALO was verified in GL261 and U87 GBM cells, corroborating the aforementioned conclusions. But the expression changes of relative protein similar to the referred report [24] were not verified (data not shown). Additionally, marked induction of apoptosis was found only at a relatively high ALO concentration, with a minimum of 0.25 mM in GL261 and 0.50 mM in U87. The effect was minimal at a low concentration even when the time course was extended to 48 h. Therefore, we conclude that ALO’s effects on cell cycle arrest and apoptosis are likely secondary effects.

ALO has dual hydrophilic and hydrophobic characteristics and a small molecular weight, allowing it to cross the cell membrane and enter the cytoplasm. we assumed that ALO could cross the cell membrane and enter the cytoplasm. We hypothesized that ALO could target various organelles, such as mitochondria, the ER, and autophagosomes. Through TEM observations, we confirmed changes in these organelles after ALO treatment. ALO coupled with a fluorescent dye is an inexpensive and convenient method for tracing its localization, similar to mature peptide-tracing technology [25]. So in this study, we used fluorescent dye-coupled ALO to show that ALO directly targets lysosomes. However, there remain some unfavourable factors due to the similar molecular properties of ALO and the dye, which can be addressed in further research to optimize outcomes and provide more convincing conclusions. One is that the coupling product was not purified in this research due to the difficulty in doing so, and the free dye caused interference. Another is the uncertainty about changes in the biological function of ALO after coupling. Thus, we further confirmed a reduction in lysosome function after ALO treatment by the acidic conditions.

Autophagy has drawn our attention because both the product of autophagosomes and ALO target lysosomes directly. The assessment of autophagy relies on the comprehensive application of various experimental methods, with no individual strategy perfect for all situations [26]. ALO has been reported to induce autophagy in thyroid cancer cells [19], but inhibit it in lung cancer cells [18], possibly indicating tissue specificity. In this study, ALO treatment led to the inhibition of late autophagy, as shown by the dramatic accumulation of the autophagy marker protein LC3B and increased P62 expression in GL261 cells, but no change in U87 cells. This inhibition was further verified by an autophagic flux analysis and the antagonistic effect of ALO on CQ, rather than leupeptin. LC3B accumulation after ALO treatment was also verified in vivo. In classical autophagy, LC3B transforms the nonlipidated (LC3B-I) to the lipidated (LC3B-II) form [26]. Interestingly, the increase in LC3B expression after ALO treatment was due to both slower degradation rate and higher transcription levels. Furthermore, inhibiting upstream autophagy protein activity with 3-MA treatment reduced the killing effect of ALO, suggesting that ALO’s core function may be the blockade of autophagosome-lysosome fusion under specific conditions, which requires further investigation.

Cytoplasmic vacuolization is a complex morphological phenomenon that leads to rapid cell death and is triggered via different pathways in cultured mammalian cells [27]. It can be induced by various treatments, including exposure to low-molecular-weight compounds [28]. We found that cytoplasmic vacuoles appeared in GBM cells treated with a low concentration of ALO for as few as 8 h, making it the first time this phenomenon has been discovered in GBM. Although previous studies have shown that geldanamycin and bortezomib cotreatment can cause ER-derived cytoplasmic vacuolization [29], we did not verify this effect in our study.

Cytoplasmic vacuolization is one of the unique features of paraptosis [28, 30], which is a recently described form of programmed cell death that has the potential to overcome tumour drug resistance [31], as a critical adjutant of mature cell death pathways, such as apoptosis and necrosis. Paraptosis has been defined as a form of cell death that is independent of caspase activation [32, 33], but it has also been reported in conjunction with autophagy and apoptosis [34–36]. Other classical but unnecessary features of paraptosis include ER dilation and mitochondrial swelling, ER stress and inhibition by CHX, MAPK signalling pathway activation, proteostasis disruption, and ion and redox homeostasis alteration [30]. In our study, most of these features were verified in GBM cells after ALO treatment, but the changes were not consistent across different cell lines.

Our findings suggest that the inhibition of late autophagy induced an imbalance in the synthesis and decomposition of proteins, leading to ER stress and ROS generation. This imbalance could be reversed by inhibiting transcription or translation, and it resulted in paraptosis induction after ALO treatment. ALO treatment also induced significant ER stress in vivo and appeared to exert a synergistic effect with TMZ, which may be explained by the additional effects of this combination mediated through different mechanisms.

In conclusion, this study demonstrated that ALO targets lysosomes through a unique labeling approach, and its cytotoxic effects in GBM are mediated by inhibiting late-stage autophagy, inducing apoptosis, and triggering paraptosis. These findings suggest that ALO has therapeutic potential for GBM treatment.

## Materials and methods

### Cell lines and reagents

The murine GL261 (RRID: CVCL_Y003) glioma cell line, which has been frequently used to assess experimental GBM therapies [37], was purchased from the Chinese National Collection of Authenticated Cell Cultures, and the patient-derived U87 (RRID: CVCL_3429) and A172 (RRID: CVCL_0131) GBM cell lines were bought from the American Type Culture Collection. A normal human astrocyte (HA) cell line (RRID: CVCL_B5WG) was provided by the Institute of Basal Medical Sciences. All the cells were cultured in high-glucose DMEM (Gibco) supplemented with 10% foetal bovine serum (FBS; S030421, NEWZERUM) and 1% penicillin-streptomycin (HyClone). The compounds used in this study included ALO (HY-13,536, MedChemExpress [MCE]), necrostatin-1 (HY-15,760, MCE), Z-VAD-FMK (HY-16658B, MCE), cycloheximide (CHX; #2112, Cell Signaling Technology [CST]), actinomycin D (ActD; M4881, AbMole), chloroquine (CQ; HY-17,589 A, MCE), 3-methyladenine (3-MA; HY-19,312, MCE), leupeptin hemisulfate (HY-18,234 A, MCE), and TMZ (PHR1437, Sigma).

### Cell counting kit-8 (cck-8) assay

A total of 10^4^ cells/well were plated in a 96-well plate and incubated for approximately 12 h. Then, different concentrations of ALO (0, 0·125, 0.25, 0.50, and 1.0 mM) were added, and the cells were incubated for another 24 h. After the medium was replaced with 10% cck-8 reagent for 1 h, the optical density (OD) value was measured at 450 nm with a full-wavelength microplate reader (Thermo). For certain experiments, cells were treated with different inhibitors (necrostatin-1, Z-VAD, 3-MA, CQ, or leupeptin) or protein expression inhibitors (ActD or CHX) 2 h before treatment with ALO, and then cocultured for another 24 h, before a cck-8 test assay was performed.

### Clone formation assay

A total of 10^3^ single cells/well were plated evenly in a 6-well plate. ALO was added after 24 h (ALO concentration gradient: 0, 0.25, and 0.50 mM for U87 and A172; 0, 0.125, and 0.25 mM for GL261), and then the cells were incubated for another 7 days. The cells were fixed with 4% paraformaldehyde and stained with crystal violet staining solution. Photos of the colonies were captured with a camera, and the number was counted with ImageJ software.

### Cell cycle analysis

When cells reached 70% confluency in a 6-well plate, cells were synchronized by replacing the medium with fresh DMEM without FBS and incubated for 12 h. Next, the cells were exposed to ALO (concentration gradient: 0, 0.125, 0.25, and 0.50 mM; diluted in complete DMEM) and incubated for 24 h, followed by digestion and collection. After fixation with 1 ml precooled 70% ethanol for 12 h, the cell samples were stained with propidium iodide (PI), and assayed with a cell cycle and apoptosis analysis kit (C1052, Beyotime). The signals were detected by flow cytometry (BECKMAN CytoFlex) in the phycoerythrin (PE) channel and analysed with FlowJo V10 software.

### Apoptosis analysis

After cells were treated with different concentrations of ALO for specific periods of time (from 8 to 48 h) in a 6-well plate, both adherent cells digested and the cells floating in the supernatant were collected. Then approximately 50,000 cells were stained with Annexin V and PI (C1062, Beyotime), and flow cytometry was performed, followed by FlowJo analysis.

### Quantitative protein expression analysis

Total protein was extracted from cells cultured in vitro or in brain tumours of murine models with RIPA lysis buffer (supplemented with phenylmethylsulfonyl fluoride [PMSF] and phosphatase inhibitor cocktails) and examined by western blotting (WB) as previously described [38]. The antibodies used for WB are listed in Table S1. β-actin or GAPDH was used as the housekeeping gene.

### TEM

Samples of precipitated cells (after 0.25 mM ALO treatment for 24 h or the negative control [NC]) were harvested and fixed in TEM fixative (G1102, ServiceBio) at 4 ℃ for at least 24 h. Cell dehydration, embedding, section slicing, staining, and image capturing were completed by KeyGen Biotech. We focused on changes in the mitochondria, endoplasmic reticulum (ER), and autophagosomes.

### Organelle-specific fluorescent probe staining

Lyso-Tracker Red (C1046, Beyotime) or Lyso-Tracker Green (40738ES50, Yeasen), ER-Tracker Green (C1042, Beyotime), and Mito-Tracker Green (C1048, Beyotime) were applied to label lysosomes, the ER, and mitochondria in live cells, respectively. Lyso-Tracker was diluted in DMEM by 1:10,000, and Mito-Tracker by 1:5,000. ER-Tracker was diluted in the matching diluent by 1:3,000. These dyes were incubated with cells for 1 h at room temperature. Cells were then observed and imaged under a fluorescence microscopy.

### 5-(6)-TAMRA-SE-labelled ALO localization assessment

A fluorescent dye, 5-(6)-TAMRA-SE, was used to label ALO (1:1 by volume) after 1 h of co-incubation at room temperature as previously described [25]. In this experiment, ALO was diluted in NaHCO_3_ solution, to maintain a pH of around 8.5. Because the function of ALO is inhibited by acidic environments, the quenching step performed by adding acetic acid was omitted. The cells were treated with fluorescent-labelled ALO for 4–12 h before stained by the other probes.

### Quantitative mRNA expression analysis

Total RNA was extracted from cells cultured in vitro with an animal RNA isolation kit (R0027, Beyotime), and quantified with a full-wavelength microplate reader. A reverse transcription reaction and real-time quantitative PCR were completed with kits (R312-01 and Q431, Vazyme) following the manufacturer’s instructions on a BIO-RAD S1000 Thermal Cycler System and an Agilent AriaMx qPCR instrument, respectively. The primers used for qPCR were synthesized by Generay Biotechnology, and the sequences are shown in Table S2. β-actin was used as the housekeeping gene. The comparative CT (2^−ΔΔCT^) method was used to compare relative gene expression levels among different groups.

### Adenovirus and siRNA transfection

An mRFP-GFP-LC3 fusion protein-expressing adenovirus for autophagy study (Hanbio Biotechnology) was used to monitor the formation and degradation of autophagosomes. Transfection was performed in the GL261 and A172 cell lines at a multiplicity of infection (MOI) of 10 following the manufacturer’s instructions. Two siRNA sequences targeting m-LC3B (shown in Table S3) were used for gene expression knockdown with the transfection reagent (jet PRIME® Polyplus) under the standard process, and the final concentration of siRNA was 10 nM. All the subsequent experiments were performed within 48 h after transfection.

### ROS assay

Cells were treated with different concentrations of ALO for 12 h in a 12-well plate, and DCFH-DA probes, diluted in basic DMEM (1:5000), were applicated to measure ROS generation. The probes were co-incubated with the cells for 20 min. After gently washed with DMEM, the cells were observed under a fluorescence microscope. Rosup was added at a concentration of 50 µg/ml as the positive control.

### Intracranial stereotactic allograft glioma models in vivo

Six-week-old C57BL/6 male mice weighing 20–25 g were purchased from GemPharmatech, and divided randomly into each group. Approximately 2 × 10^6^ GL261 cells with stable luciferase expression were digested and adjusted to a volume of 10 µl, then injected into the brain of each mouse. The injection location was 0.5 mm anterior and 2.5 mm lateral from the bregma, at a depth of 4 mm. Other procedures were performed according to the instructions [39]. The day that the models were established was recorded as day 0. Mouse body weight was measured every 7 days after cell injection, and live imaging was performed with luciferin (P104C, Promega) injected 10 min in advance on days 7 and 21. Both ALO and TMZ treatments were administered by intraperitoneal injection. The solvents were added to 50 mM ALO dissolved in ddH_2_O or 50 mg/ml TMZ in DMSO sequentially as followed: 30% PEG 400, 0.5% Tween 80, and 5% propylene glycol, or 30% PEG 400, 5% Tween 80, and 60% saline. ALO at a dose of 50 mg/kg was administered every other day (on days 7, 9, 11, 13, 15, 17, and 19; 7 times in total) to the mice in the ALO monotherapy group. In the TMZ-and-ALO cotherapy group, 25 mg/kg TMZ and 50 mg/kg ALO were given every third day, and these two drugs were administered on different days to maintain volume-control (TMZ: on days 7, 10, 13, 16, and 19; ALO: on days 8, 11, 14, 17, and 20; 5 times each). The control group was treated with the corresponding solvent.

### IHC

To remove the blood from the circulatory system, fully anaesthetized mice were perfused with normal saline through the right ventricle. Then, whole brains with tumours were removed and incubated in 4% paraformaldehyde for fixation. Paraffin embedding, sectioning, and IHC staining (with LC3B antibody [CST Cat#83,506, RRID:AB_2800018], ki-67 antibody [CST Cat#9027, RRID:AB_2636984], and Tunel method) were completed by Servicebio.

### Statistical analyses

All the experiments were performed in three independent biological replicates. The data are presented as the means ± SD. Statistical analysis and graphing were performed using GraphPad Prism 8.0 software. Differences between two groups were statistically analysed by Student’s t test, and multiple comparisons were analysed by one-way ANOVA followed by Tukey’s test for two-by-two comparisons. Differences were considered statistically significant when *p* < 0.05.

### Schematic diagram

The schematic diagram (Fig. 7) was generated with Figdraw (export ID: OSUIU48e8c) and Reactome [40].

### Supplementary Information

**Additional file 1: Figure S1.** ALO inhibited late autophagy in glioma cells in vitro. (a) Tracking of the autophagic flux following ALO treatment dynamically by introducing a tandem mRFP-GFP-LC3 adenovirus into U87, with EBSS treatment group as the autophagic positive control. (b) Expression of autophagy-related proteins, LC3B and p62, exposed to ALO in GL261 tested by WB (*n* = 3). (c) Transcriptional expression of LC3B, after ALO treatment in U87 tested by qPCR (*n* = 3). (d) Expression of LC3B by WB, exposed to PI3K inhibitor, 5 mM 3-MA, with or without ALO in U87, to ensure the inhibitory effect on the autophagic pathway (*n* = 3). (e) Cell viability of U87 exposed to ALO, pre-treated with 3-MA by cck-8 test (*n* = 4). (f) Protein expression of LC3B, exposed to 10 μM CQ, with or without ALO in U87 tested by WB (*n* = 3).  Data are presented as mean ± SD, *p< 0.05, **p < 0.01, ***p < 0.001, ****p < 0.0001, ns means not significant. ALO, aloperine; NC, negative control; WB, western blot; EBSS, Earle's Balanced Salt Solution; CQ, Chloroquine; 3-MA, 3-Methyladenine; WT, wildtype. **Figure S2**. ALO targeted lysosomes directly and weakened its acidic conditions in GBM cells in vitro. (a) Tamra-se coupled ALO and ER-targeted fluorescent probe (green) colocalization analysis in GL261 and A172 (*n* = 3). (b) Tamra-se coupled ALO and mitochondria-targeted fluorescent probe (green) colocalization analysis in GL261 and A172 (*n* = 3). (c) Protein expression of LC3B, exposed to Leupeptin, with or without ALO in GL261 and U87 tested by WB (*n* = 3). Data are presented as mean ± SD, ***p< 0.001, ****p < 0.0001, ns means not significant. ALO, aloperine; NC, negative control; WB, western blot. **Figure S3. **ALO induced paraptosis in GBM cells in vitro. (a) Expression of ER stress-related proteins exposed to ALO in U87 tested by WB (*n* = 3). (b) Activation of MAPK pathways exposed to ALO in GL261 tested by WB (*n* = 3). (c) Cell viability of U87 exposed to ALO by cck-8 test, pretreated with 2 μM ActD or 2 μg/ml CHX for 2 h (*n* = 4). (d) Cytoplasmic vacuolations were observed under the light microscope after ALO treatment in A172 with or without CHX pretreatment for 2 h. Cell viability of exposed to ALO by cck-8 test, pretreated with 2 μM ActD or 2 μg/ml CHX for 2 h (*n* = 4). Data are presented as mean ± SD, *p< 0.05, **p < 0.01, ***p < 0.001, ****p < 0.0001, ns means not significant. ActD, Actinomycin D; ALO, aloperine; CHX, cycloheximide; ER, endoplasmic reticulum; NC, negative control; WB, western blot. **Table S1.** Antibodies applied in western blot. **Table S2.** Primer sequences applied in qPCR. **Table S3.** siRNA sequences.

## Data Availability

All the data and materials included in this study have been fully explained in the manuscript and Additional files.
