# Supplementary material for: Aloperine targets lysosomes to inhibit late autophagy and induces cell death through apoptosis and paraptosis in glioblastoma
Source: Mol Biomed. 2023 Nov 17;4:42. doi: 10.1186/s43556-023-00155-x (PMC10656413; doi:10.1186/s43556-023-00155-x)
Supplement: Supplementary file 1 — Additional file 1: Figure S1. ALO inhibited late autophagy in glioma cells in vitro. (a) Tracking of the autophagic flux following ALO treatment dynamically by introducing a tandem mRFP-GFP-LC3 adenovirus into U87, with EBSS treatment group as the autophagic positive control. (b) Expression of autophagy-related proteins, LC3B and p62, exposed to ALO in GL261 tested by WB (n = 3). (c) Transcriptional expression of LC3B, after ALO treatment in U87 tested by qPCR (n = 3). (d) Expression of LC3B by WB, exposed to PI3K inhibitor, 5 mM 3-MA, with or without ALO in U87, to ensure the inhibitory effect on the autophagic pathway (n = 3). (e) Cell viability of U87 exposed to ALO, pre-treated with 3-MA by cck-8 test (n = 4). (f) Protein expression of LC3B, exposed to 10 μM CQ, with or without ALO in U87 tested by WB (n = 3). Data are presented as mean ± SD, *p< 0.05, **p < 0.01, ***p < 0.001, ****p < 0.0001, ns means not significant. ALO, aloperine; NC, negative control; WB, western blot; EBSS, Earle's Balanced Salt Solution; CQ, Chloroquine; 3-MA, 3-Methyladenine; WT, wildtype. Figure S2. ALO targeted lysosomes directly and weakened its acidic conditions in GBM cells in vitro. (a) Tamra-se coupled ALO and ER-targeted fluorescent probe (green) colocalization analysis in GL261 and A172 (n = 3). (b) Tamra-se coupled ALO and mitochondria-targeted fluorescent probe (green) colocalization analysis in GL261 and A172 (n = 3). (c) Protein expression of LC3B, exposed to Leupeptin, with or without ALO in GL261 and U87 tested by WB (n = 3). Data are presented as mean ± SD, ***p< 0.001, ****p < 0.0001, ns means not significant. ALO, aloperine; NC, negative control; WB, western blot. Figure S3. ALO induced paraptosis in GBM cells in vitro. (a) Expression of ER stress-related proteins exposed to ALO in U87 tested by WB (n = 3). (b) Activation of MAPK pathways exposed to ALO in GL261 tested by WB (n = 3). (c) Cell viability of U87 exposed to ALO by cck-8 test, pretreated with 2 μM ActD or 2 [file 43556_2023_155_MOESM1_ESM.docx]

**Title:** **Aloperine Targets Lysosomes to Inhibit Late Autophagy and Induces Cell Death through Apoptosis and Paraptosis in Glioblastoma**

**Running title: Aloperine Targets Lysosomes in Glioblastoma**

**Authors:** Ting Tang^1,3*^, Hui Liang^2,3*^, Wuting Wei^3^, Yanling Han^3^, Liang Cao^4^, Zixiang Cong^3^, Shiqiao Luo^5^, Handong Wang^3,6#^, Meng-Liang Zhou^3#^

^1^Department of Neurosurgery, Xuanwu Hospital Capital Medical University, Beijing, P.R. China.

^2^Department of Neurosurgery, The First Affiliated Hospital, Guangxi Medical University, Nanning, P.R. China.

^3^Department of Neurosurgery, Affiliated Jinling Hospital, Medical School of Nanjing University, Nanjing, P.R. China.

^4^Department of Medical Oncology, Affiliated Chuzhou Hospital of Anhui Medical University, The First People's Hospital of Chuzhou, Chuzhou, P.R. China.

^5^Department of Neurosurgery, Affiliated Jinling Hospital, Nanjing Medical University, Nanjing, P.R. China.

^6^Department of Neurosurgery, Benq Medical Center, Nanjing Medical University, Nanjing, China

# Correspondence: Handong Wang, [njhdwang@hotmail.com](mailto:njhdwang@hotmail.com);

Meng-Liang Zhou, mengliangzhou@yahoo.com.

^*^ The authors contributed equally to this work.


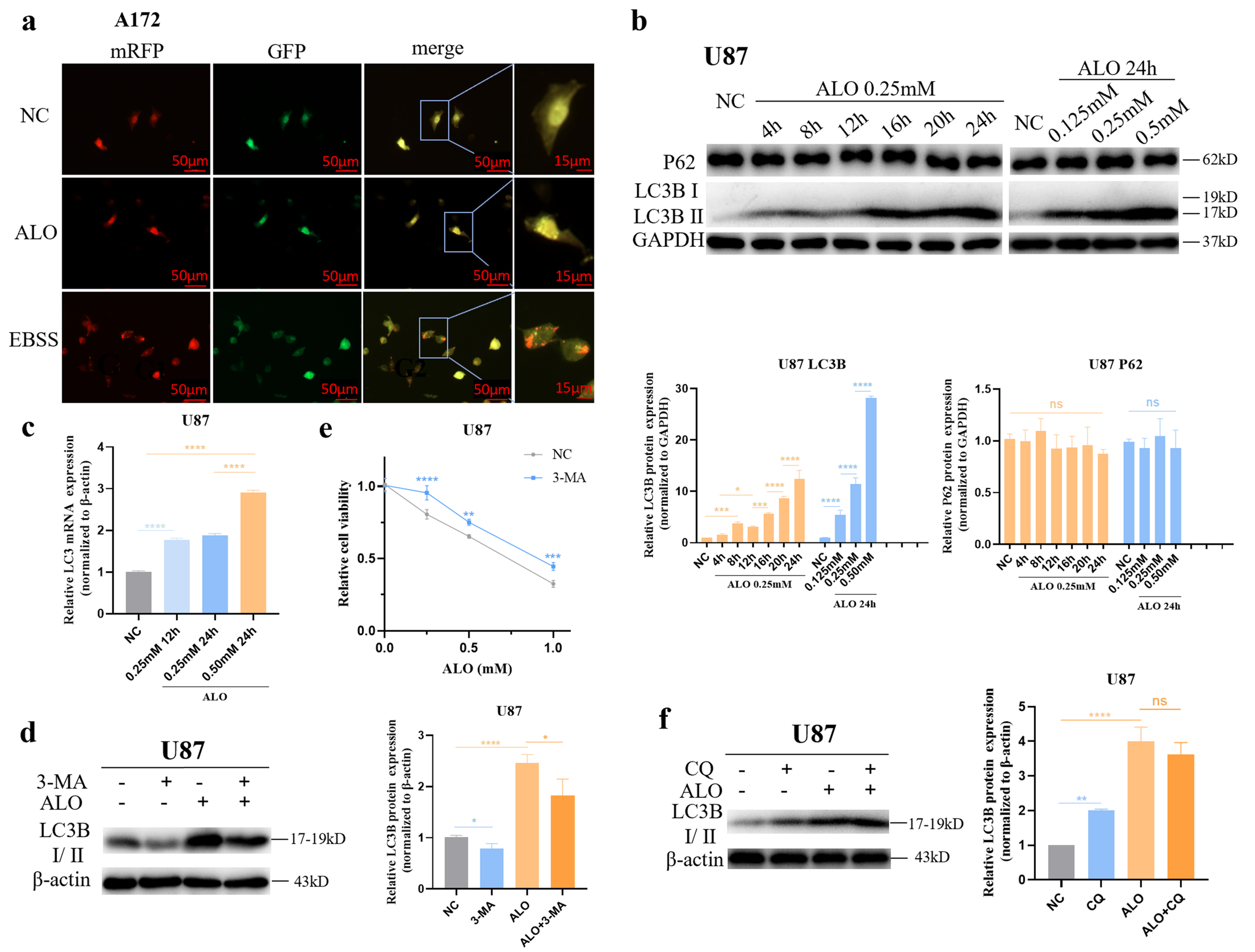
**Figure S1. ALO inhibited late autophagy in glioma cells in vitro. (a)** Tracking of the autophagic flux following ALO treatment dynamically by introducing a tandem mRFP-GFP-LC3 adenovirus into U87, with EBSS treatment group as the autophagic positive control. **(b)** Expression of autophagy-related proteins, LC3B and p62, exposed to ALO in GL261 tested by WB (n = 3). **(c)** Transcriptional expression of LC3B, after ALO treatment in U87 tested by qPCR (n = 3). **(d)** Expression of LC3B by WB, exposed to PI3K inhibitor, 5 mM 3-MA, with or without ALO in U87, to ensure the inhibitory effect on the autophagic pathway (n = 3). **(e)** Cell viability of U87 exposed to ALO, pre-treated with 3-MA by cck-8 test (n = 4). **(f)** Protein expression of LC3B, exposed to 10 μM CQ, with or without ALO in U87 tested by WB (n = 3). Data are presented as mean ± SD, **p* < 0.05, ***p* < 0.01, ****p* < 0.001, *****p* < 0.0001, ns means not significant. ALO, aloperine; NC, negative control; WB, western blot; EBSS, Earle's Balanced Salt Solution; CQ, Chloroquine; 3-MA, 3-Methyladenine; WT, wildtype.


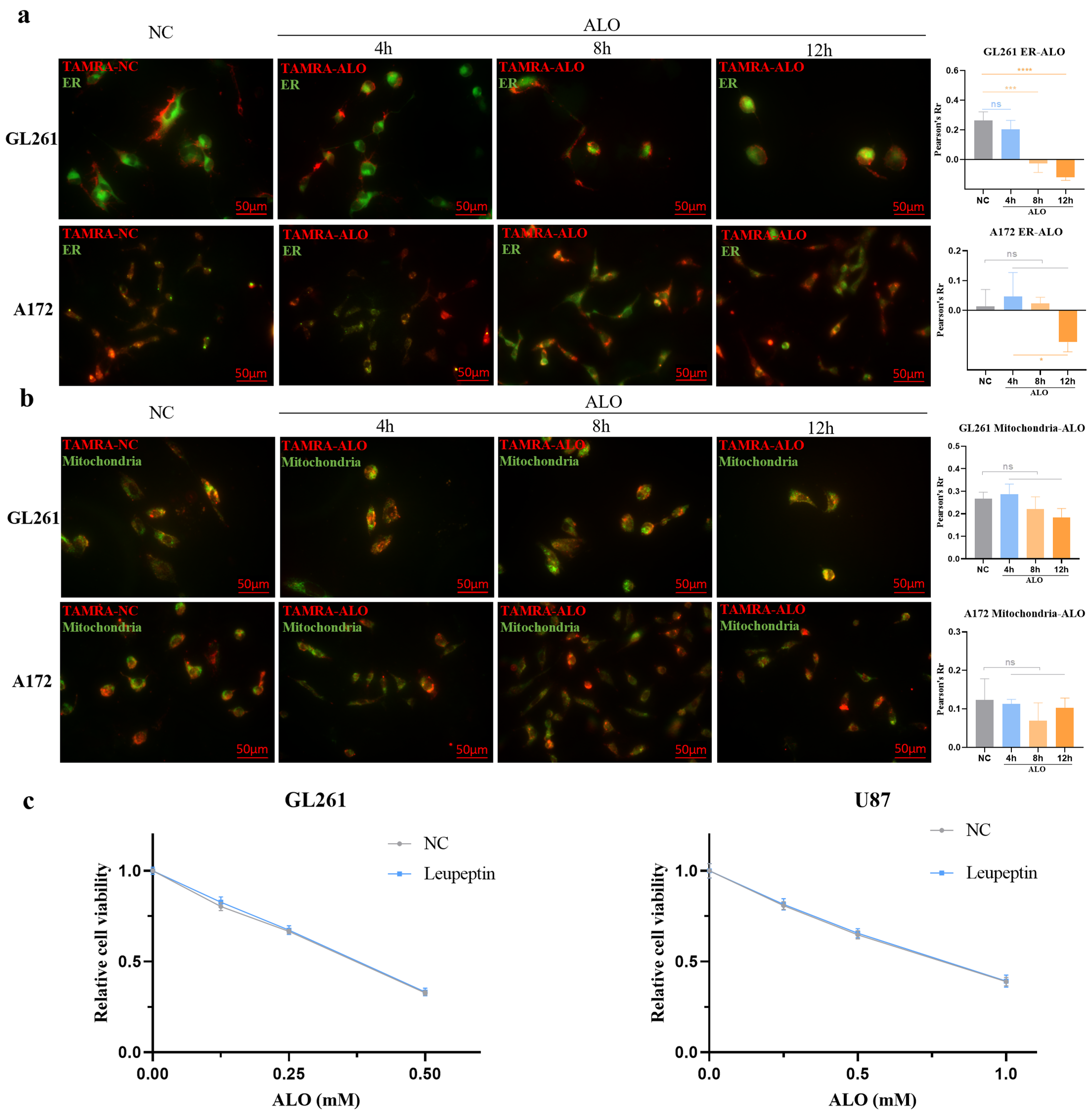


**Figure S2. ALO targeted lysosomes directly and weakened its acidic conditions in GBM cells in vitro.** **(a)** Tamra-se coupled ALO and ER-targeted fluorescent probe (green) colocalization analysis in GL261 and A172 (n = 3). **(b)** Tamra-se coupled ALO and mitochondria-targeted fluorescent probe (green) colocalization analysis in GL261 and A172 (n = 3). (c) Protein expression of LC3B, exposed to Leupeptin, with or without ALO in GL261 and U87 tested by WB (n = 3). Data are presented as mean ± SD, ****p* < 0.001, *****p* < 0.0001, ns means not significant. ALO, aloperine; NC, negative control; WB, western blot.


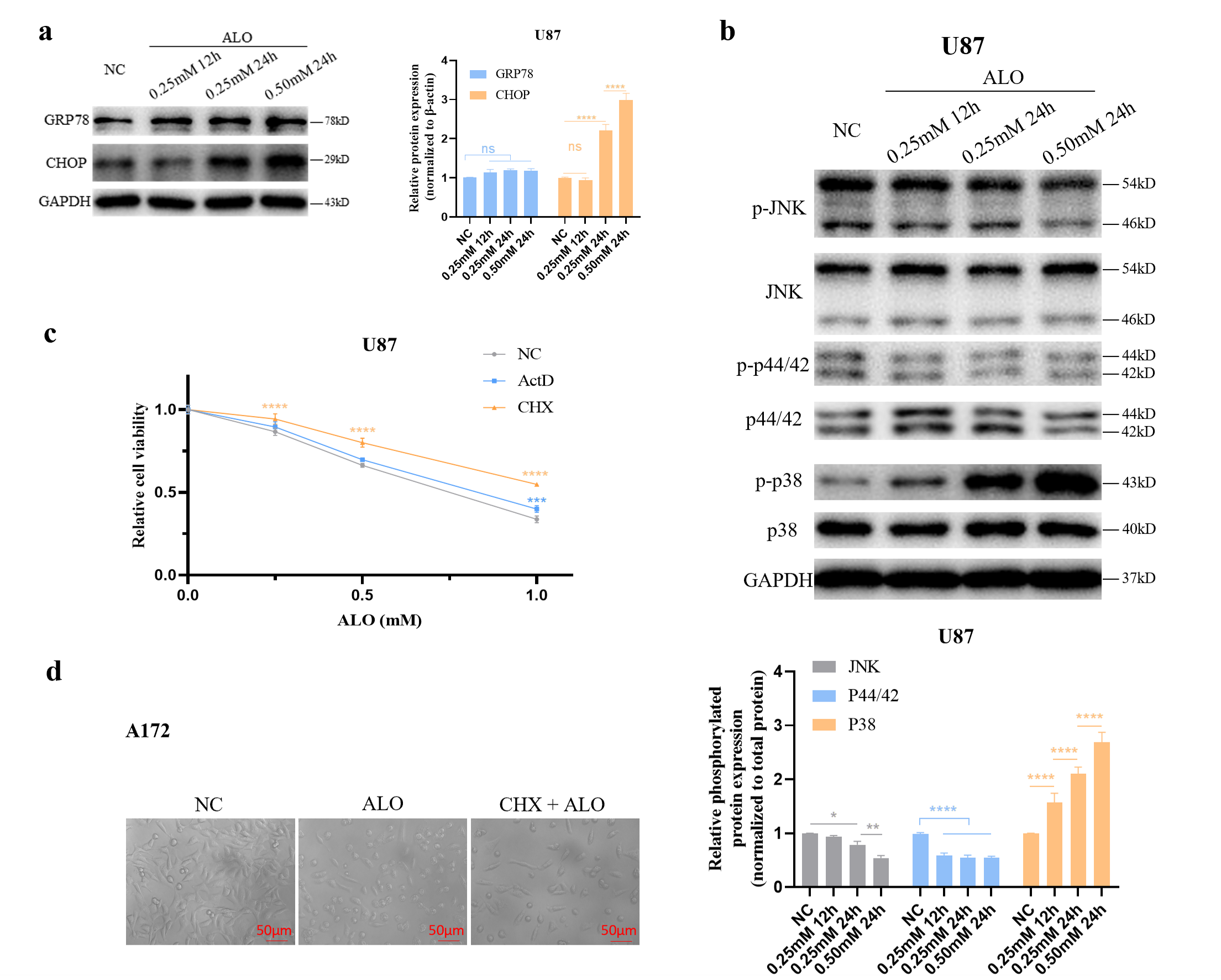


**Figure S3. ALO induced paraptosis in GBM cells in vitro. (a)** Expression of ER stress-related proteins exposed to ALO in U87 tested by WB (n = 3). **(b)** Activation of MAPK pathways exposed to ALO in GL261 tested by WB (n = 3). **(c)** Cell viability of U87 exposed to ALO by cck-8 test, pretreated with 2 μM ActD or 2 μg/ml CHX for 2 h (n = 4). **(d)** Cytoplasmic vacuolations were observed under the light microscope after ALO treatment in A172 with or without CHX pretreatment for 2 h. Cell viability of exposed to ALO by cck-8 test, pretreated with 2 μM ActD or 2 μg/ml CHX for 2 h (n = 4). Data are presented as mean ± SD, **p* < 0.05, ***p* < 0.01, ****p* < 0.001, *****p* < 0.0001, ns means not significant. ActD, Actinomycin D; ALO, aloperine; CHX, cycloheximide; ER, endoplasmic reticulum; NC, negative control; WB, western blot.

Table S1 Antibodies applied in western blot.

| **Antibody** | **Supplier** | **Catalog number** | **Source** | **Dilution** |
| --- | --- | --- | --- | --- |
| Anti-CyclinD1 antibody | CST | #2978 | Rabbit | 1:1000 |
| Anti-CDK6 antibody | CST | #3136 | Mouse | 1:1000 |
| Anti-CDK4 antibody | CST | #12790 | Rabbit | 1:1000 |
| Anti-MAP1LC3B antibody | NOVUS | NB600-1384 | Rabbit | 1:1000 |
| Anti-P62 antibody | Servicebio | GB11239-1 | Rabbit | 1:500 |
| Anti-GRP78 antibody | Servicebio | GB11098 | Rabbit | 1:1000 |
| Anti-CHOP antibody | Proteintech | 15204-1-AP | Rabbit | 1:1000 |
| Anti-phospho-p44/42 MAPK (Erk1/2) (Thr202/Tyr204) antibody | CST | #4370 | Rabbit | 1:1000 |
| Anti-p44/42 MAPK antibody | CST | #4695 | Rabbit | 1:1000 |
| Anti-JNK1 + JNK2 + JNK3 (phospho T183+T183+T221) antibody | Abcam | ab124956 | Rabbit | 1:1000 |
| Anti-SAPK/JNK antibody | CST | #9252 | Rabbit | 1:1000 |
| Anti-phospho-p38 MAPK (Thr180/Tyr182) antibody | CST | #4511 | Rabbit | 1:1000 |
| Anti-p38 MAPK antibody | CST | #8690 | Rabbit | 1:1000 |
| Anti-caspase3 antibody | Proteintech | 19677-1-AP | Rabbit | 1:1000 |
| Anti-cleaved-caspase3 antibody | CST | #9661 | Rabbit | 1:1000 |
| Anti-GAPDH antibody | Bioworld | AP0063 | Rabbit | 1:5000 |
| Anti-β-actin antibody | Bioworld | AP0060 | Rabbit | 1:5000 |
| Anti-rabbit IgG-HRP antibody | HuaBio | HA1001 | Goat | 1:10000 |
| Anti-mouse IgG-HRP antibody | HuaBio | HA1006 | Goat | 1:10000 |

| **Primer Name** | **Primer Sequence** |
| --- | --- |
| h-MAP1LC3B F | 5’- GACCCTGGAGAAAGAGTGG -3’ |
| h-MAP1LC3B R | 5’- TCCGTAACAACACAGGCA -3’ |
| h-β-actin F | 5’- ATTGCCGACAGGATGCAGAA -3’ |
| h-β-actin R | 5’- GCTGATCCACATCTGCTGGAA -3’ |
| m-MAP1LC3B F | 5’- ATGGTGCGATCAGTAAGGA -3’ |
| m-MAP1LC3B R | 5’- CAGGTTCGTTGTGCCTTT -3’ |
| m-β-actin F | 5’- CCTCACTGTCCACCTTCC -3’ |
| m-β-actin R | 5’- GGGTGTAAAACGCAGCTC -3’ |

Table S2 Primer sequences applied in qPCR.

Table S3 siRNA sequences

| **siRNA** | **Sequence（sense, 5'-3'）** |
| --- | --- |
|  |  |
| si-LC3B-1 | GCAGCUCAAUGCUAACCAATT |
| si-LC3B-2 | GCUUCCUGUACAUGGUUUATT |
| si-NC | UUCUCCGAACGUGUCACGUTT |
